# Number of addictive substances used related to increased risk of unnatural death: A combined medico-legal and case-record study

**DOI:** 10.1186/1471-244X-9-48

**Published:** 2009-08-04

**Authors:** Louise Brådvik, Mats Berglund, Arne Frank, Anna Lindgren, Peter Löwenhielm

**Affiliations:** 1 Clinical Sciences, Lund, Sweden; 2 Clinical Alcohol Research, Malmö, Sweden; 3 Centre for Mathematical Sciences, Lund, Sweden; 4 Forensic Medicine, Lund, Sweden

## Abstract

**Background:**

Substance use disorders have repeatedly been found to lead to premature death, i.e. drug-related death by disease, fatal intoxications, or trauma (accidents, suicide, undetermined suicide, and homicide). The present study examined the relationship between multi-drug substance use and natural and unnatural death.

**Methods:**

All consecutive, autopsied patients who had been in contact with the Addiction Centre in Malmö University Hospital from 1993 to 1997 inclusive were investigated. Drug abuse was investigated blindly in the case records and related to the cause of death in 387 subjects.

**Results:**

Every substance apart from alcohol used previously in life added to the risk of unnatural death in a linear way. There were independent increased risks of fatal heroin overdoses or undetermined suicide. Death by suicide and violent death were unrelated to additional abuse.

**Conclusion:**

The number of drugs used was related to an increased risk of unnatural death by undetermined suicide (mainly fatal intoxications) and heroin overdose.

## Background

Substance use disorders, either alone or in combination with other psychiatric disorders, have repeatedly been found to lead to premature death, i.e. drug-related death by disease, fatal intoxications, or trauma (accidents, suicide, undetermined suicide, and homicide) [1]. In medico-legal practice, distinction is made between natural and unnatural death, where natural death is caused by disease only. Unnatural deaths are classified as 'accident', 'suicide' or 'homicide' in order to meet the demands of Swedish death statistics. Finally, in suicidology the degree of intent in self-inflicted death is studied with concepts like "self-inflicted unintentional death", "self-inflicted death with undetermined intent", and "suicide" [2].

A total of 63% of the drug-related deaths were registered as unnatural deaths in a Danish study [3]. In medico-legal autopsy studies, a positive blood alcohol test has been found in about 40% of all unnatural deaths [4,5].

Suicide is commonly attributed to substance use. Overall, substance use (alcoholism included) is found in 25–55% of suicides, a rate far in excess of its prevalence in the adult population [6]. Increased suicide rates have been reported for alcohol dependence and abuse, a combination of alcohol and legal drugs, opioid dependence and abuse, and also cannabis dependence according to reviews [7,8]. Positive findings of alcohol in blood samples taken at autopsy occur in about 40% of suicide victims. [9-11].

Fatal intoxications are common amongst substance users. Those could be unintentional, undetermined suicide or suicide. The rates of positive alcohol in blood at autopsy in undetermined suicides are similar to those found in suicide, around 40% [10,4]. Alcohol, sedatives, such as benzodiazepines, and narcotics such as heroin, are commonly found in fatal intoxications [11-16]. A medico-legal study of fatal intoxications in drug addicts in the five Nordic countries in 2002 [17] has revealed that heroin/morphine was the single most frequently encountered main intoxicant; frequently seen substances in addition to the main intoxicant were amphetamine, tetrahydrocannabinol (THC), benzodiazepines and ethanol. Heroin overdoses are fatal intoxications, and are a major contributor to premature death among heroin users [18-22]. A combination of drugs is frequently found in fatal intoxications [13,17,23,24], with a previous Swedish study showing an average of 3.8 drugs at autopsy in deceased addicts in Sweden [25].

Victims of violent death by accidents and homicide often show positive concentrations for alcohol and drugs [4,26-33]. Alcohol and other drugs are strongly associated with violent death resulting from motor vehicle crashes, and in victims of all other types of trauma mortality, specifically those victims of gunshot wounds, burns, stabbings, electricity, and falls.

The present study examines a consecutive sample of cases autopsied for medico-legal reasons. All of these cases were former inpatients or outpatients at Malmö University Hospital. Independent information on these cases, including previous addiction and causes of death, was also obtained. The aim of the present study was to relate different types of death to alcohol abuse and number of additional illegal and legal drugs.

## Methods

A forensic examination sampling procedure was used for the present study. The procedure was carried out on all consecutive autopsies of patients who had been in contact with the Addiction Centre in Malmö University Hospital. In Sweden, forensic examination includes the majority of subjects who have died outside hospitals by suspected natural causes (disease) but with no medical history that can explain the death or by unnatural manners (trauma including homicide, suicide, undetermined suicide, and unintentional fatal intoxications). Unnatural death could be considered as accidental, self-inflicted or homicide. Death could be either violent or non-violent, as in the case of suicide. Fatal intoxications could be intentional, as in suicide, of unknown intent, as in the case of undetermined suicide, or probably unintentional, as is usually the case when the drug previously used is involved [1]. In the present study we take a particular interest in suicide and related self-inflicted death, such as undetermined suicide and unintentional drug overdoses, mainly involving heroin. The remaining cause of unnatural death was trauma, which may be secondary to risk-taking behaviour.

We chose to study natural against unnatural death. The latter was divided into undetermined suicide, heroin overdoses, suicide, and violent death.

**Figure 1:**
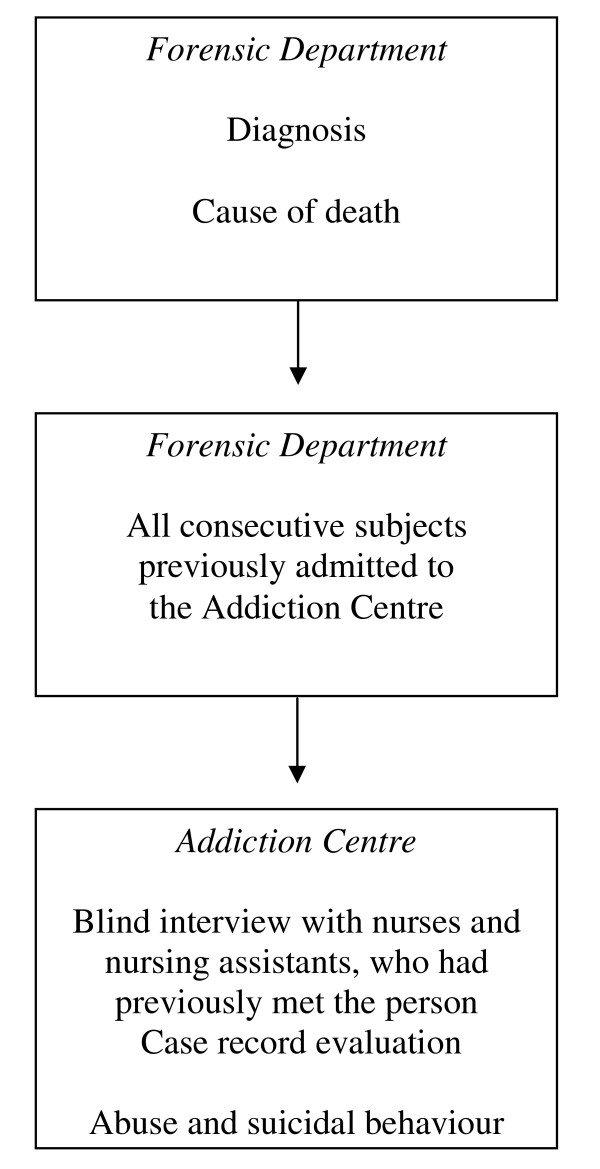
**Flow diagram showing sampling procedure**.

The procedure of the study is presented in Figure 1.

### Case record evaluations and interviews

There were 393 consecutive forensic autopsies performed on previous patients at the Department of Forensic Medicine in Lund from 1993 to 1997 inclusive. In five cases, the case records could not be found and these were excluded from the analysis, leaving 388 patients (339 men and 49 women). In one case of violent death, it could not be determined whether death was self-inflicted or caused by another person. This case (a man) was excluded from the analysis within the unnatural death group.

A pseudo-experimental design was used in which investigation was carried out within a few days of death. One member of the research team (AF) performed the interviews with the staff at the Addiction Centre. The staff included nurses and nursing assistants who had had previous contact with the patients. As the interviews were performed shortly after death, neither the interviewer nor the interviewees knew the manner of death. Thus, we managed to create a blind approach on a reasonable sample size and time of follow-up. The sampling was carried out in the 1990s, but there have been no significant changes in methodology since then.

The interviewer then evaluated the records for those who had been in- or outpatients at the Addiction Centre in Malmö University Hospital. Thus the ratings were unbiased by the knowledge of manner of death and could be considered as pseudo-prospective. The items scored were reports on type and characteristics of the addiction, information about treatment, and suicidal behaviour including suicidal thoughts.

Substance use was diagnosed according to ICD 9 and 10 [34,35] on all inpatients, and constituted 76% of the sample. The remaining 24% had been admitted as outpatients and had applied because they subjectively had a substance use problem. It is safe to conclude that they all fulfilled the criteria for alcohol dependence and/or had a drug problem. Up to 1994 all the patients treated at the Department of Clinical Alcohol Research were admitted for alcohol problems, but after that some patients may have used narcotics only, but no alcohol.

Abuse included legal and illegal drugs. The former included regular use and was divided into benzodiazepines and addictive analgesic drugs (mainly dextropropoxyphene and codeine), and the latter into opioids, cannabis, and central stimulants, mainly amphetamine. All drug use/abuse was scored independent of whether it was the main drug or not. Thus one to six drugs could be scored including alcohol and one to five apart from heroin. (Drugs that are not abused, such as antipsychotics and antidepressants, were not scored.)

### Forensic examination

After the interviews and evaluations of records, information on causes of death from the Department of Forensic Medicine was collected. The causes of death are presented in Table 1.

**Table 1:** Type of death at the Addiction Centre and Department of Forensic Medicine

Type of death	Contact with Addiction Centre#	Autopsied at the Forensic Department##	Contact with Addiction Centre ###	Age (SD)
Natural death	204	1117	18	58 (±10)
Undetermined suicide	90	238	38	52 (± 13)
Suicide	45	285	16	51 (± 11)
Heroin overdose	22	44	50*	48 (± 13)
Violent death	26	666	4	38 (± 9)

# (N = 4387)

## (N = 2350)

### (in percent)

* before 1995 6/13 (46%), 1995 and after 16/31 (52%)

Suicide was defined as: "Different manners of unnatural death have different numbers of undecided cases concerning the intent, i.e. in a hanging or a shooting it is usually easy to differentiate between a suicide or a trauma (or a crime), while for drowning, traffic accidents or intoxication it is more cumbersome. Then, circumstantial findings, such as suicide notes, expressed intent or other findings such as self-inflicted cutting of the wrist followed by drowning, are suggestive of the intent. "Undetermined suicide is defined thus: "When crime can be ruled out and it cannot be established whether the manner of death is a suicide or an accident, the manner of death is recorded as an undetermined suicide."

Heroin overdose was another cause of death, and was mostly considered unintentional [36,37]. This cause of death was not evaluated against previous abuse of heroin, as a correlation with heroin abuse was more or less a prerequisite for an unintentional fatal overdose.

Death by trauma, such as fall from height, car accident, occasional homicide, etc, was considered as violent death. All other cases were considered as natural death, i.e. when the death was caused by disease alone.

As a comparison with substances used previously in life, substances detected at autopsy were scored, including non-addictive psychopharmacologic substances.

Ethical approval was not requested for deceased persons in Sweden at that time. However, the National Board of Forensic Medicine approved the study.

### Statistics

A Pearson chi-square and a trend test were used to compare additional number of drugs and types of death.

## Results

### Type of death and contact with the Addiction Centre in Malmö

Different types of death in the forensic sample were related to previous contact with the Addiction Centre. Table 1 shows data comparing manner of death among subjects with contact with the Addiction Centre to the total subjects autopsied at the Forensic Department. The percentages of those who died by undetermined suicide and heroin overdose and who had previous contact with the Addiction Centre were each higher than for suicide and contact with the Centre. (Undetermined suicides 90/238 versus suicides 45/285, χ^2 ^= 32.86, *P *< 0.000, heroin overdoses 22/44 versus suicides 45/285, χ^2 ^= 23.93, *P *< 0.000).

A total of 157/567 (28%) of all self-inflicted fatality victims in Malmö had previous contact with the Addiction Centre.

The age at death is presented in Table 2. Death by fatal heroin overdoses occurs at a rather young age, 38 years, while those who die a natural death are oldest, 58 years. In the heroin group there was no trend towards use of more drugs in younger age groups (*P *= 0.26).

**Table 2:** Age at death

	**Age**	**Standard deviation**
Natural death	58	+/- 10

Violent death	52	+/- 13

Undetermined suicide	51	+/- 11

Suicide	48	+/- 13

Heroin overdose	38	+/- 9

### Unnatural death

**Figure 2:**
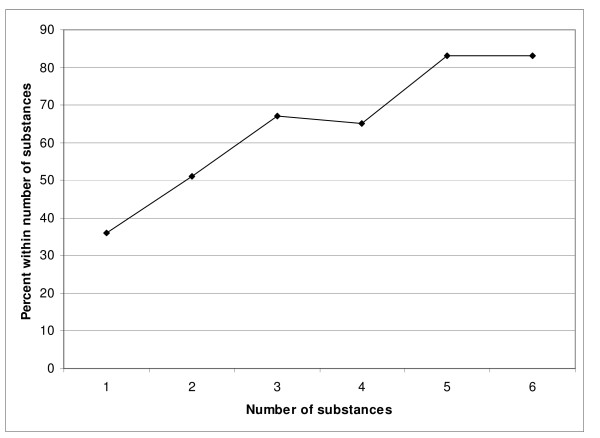
**The relationship between number of substances used risk for unnatural death**. Percent of all unnatural deaths

The number of legal and illegal psychotropic substances abused in addition to alcohol was related to unnatural types of death. There was a trend towards a higher risk for unnatural death for every additional substance used (OR = 1.64 for each substance – CI: 1.42–2.01). The increased risk is presented in Figure 2.

Heroin overdoses were included among unnatural deaths, and a relationship to additional use of drugs apart from alcohol was expected. Therefore a separate analysis was carried out for additional drugs and unnatural death apart from heroin overdoses, and the significance remained (*P *< 0.000, OR = 1.85 for each substance – CI: 1.37–2.49).

### Heroin overdoses

**Figure 3:**
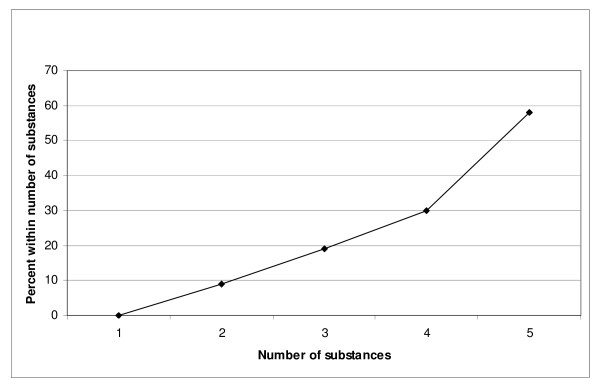
**The relationship between number of substances used and risk for heroin overdoses**. Percent of all heroin overdoses.

The risk of death by fatal heroin overdoses increased by an average of 3.5 times for every additional substance used (CI: 2.4–5.2). The increased risk is presented in Figure 3. This is highly significant (*P *< 0.000).

### Undetermined suicide

**Figure 4:**
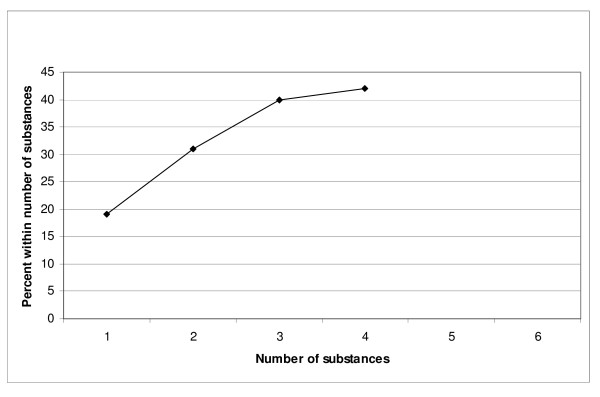
**The relationship between number of substances used and risk for undetermined suicide**. Heroin overdoses excluded. Percent of all undetermined suicides. (4 and 5 substances included together as there was only one person, an undetermined suicide, who had used 5 substances.)

As heroin overdoses were expected to make a major contribution, use of several drugs and unnatural death by other types of death were analysed after exclusion of heroin overdoses. The risk of undetermined suicide is presented in Figure 4. There is a significant trend towards increased risk of undetermined suicide for every additional substance used apart from alcohol (OR = 1.63 for each substance – CI:1.22–2.17, *P *< 0.001). A vast majority of the undetermined suicides (87/90–97%) were intoxications.

### Suicide and violent death

The risk of suicide was unrelated to number of substances used (*P *= 0.52). Furthermore, there was no increased risk of violent death when more substances were involved (*P *= 0.51).

### Number of drugs at autopsy

As a comparison with substances previously used, we compared the number of drugs identified in the toxicological analysis at autopsy. Natural deaths had lowest numbers (median = 1, mean = 0.95) followed by violent death (median = 1, mean = 1.35). Suicide and undetermined suicide showed similar numbers (both median = 2 and mean = 1.73 and = 1.90 respectively). This is an underestimate of the mean number of drugs that contributed to suicide death, as more suicides than undetermined suicides used violent methods with no drugs used at death. As expected, the highest number of substances was found in the heroin group (median = 3, mean = 3.23).

## Discussion

### Main findings

Firstly, a relatively large number of those who died by undetermined suicide and fatal heroin overdose had been in previous contact with the Addiction Centre in Malmö (38% and 50% respectively). Other unnatural and natural types of death were not as commonly associated with previous contact. Thus, it appears that substance abuse is related primarily to unnatural death by undetermined suicide and fatal heroin overdoses.

Secondly, substance use additional to alcohol was related to increased risk of unnatural death with a significant linear trend for each additional substance (ODDS ratio = 2.4). A linear trend was found for heroin overdoses and undetermined suicide. Presence of additional drugs is common in fatal heroin overdoses [37,38]. However, to our knowledge, a comparison with number of drugs used previously in life has not been made and so a linear trend with every single drug used has not been found. Non-fatal overdoses among heroin users have been shown to be related to length of heroin using career, SDS scores (Severity of Dependence Scale [39], and frequency of alcohol use [40]. However, the severity index did not specifically include the *number *of drugs, and that study concerned non-fatal overdoses only.

One study has shown that the number of substances used is more important than types of substances used in predicting *non-fatal *suicidal behaviour [41]. Disaggregation in that study showed that the effect was significant on non-planned suicide attempts but not on planned attempts among persons with suicidal ideation. The present finding, that the number of substances was related to undetermined suicide but not suicide, is compatible with the number of substances being related to unplanned but not planned attempts. Completed suicide, especially when reckoned as such, may more often be planned.

In fatal intoxications several substances are often found, as mentioned above. However, to our knowledge, a linear trend for number of additional substances *previously *used has not been shown. There was no corresponding difference in number of substances used at the time of death between suicides and undetermined suicides.

In contrast to undetermined suicide, suicide appeared to be unrelated to the number of drugs abused. Similarities between suicides and undetermined suicides have been proclaimed [42], but, on the other hand, depression has been shown to discriminate between suicide and undetermined cases in one study [43]. The discrepancy shown in the present study indicates that different mechanisms may be related to suicide and undetermined suicide. One possible explanation is that underlying depression is related to suicide, while an impulse control disorder in general may underlie poly-drug use and undetermined suicide. The latter personality disorder may also be related to heroin overdoses.

### The sample

In the present study neither the research assistant nor the staff who were interviewed was informed about the cause of death. Thus their judgement was unbiased as regards knowledge of the suicidal outcome, a problem usually inherent in a retrospective design. Consequently, the study may be considered pseudo-prospective.

All patients suffered an early death and all had contact with the alcohol clinic due to alcohol dependence and/or narcotics. In the early part of the study, only patients with an alcohol problem were included but, later, some may have a primary narcotic addiction only, which may be a source of error. Furthermore, there were no personal interviews and all data was obtained from case records, which is a limitation.

Substances used previously in life were only included if they were addictive. Thus only addictive behaviour was studied and not the possible interaction of substances used if taken simultaneously.

## Conclusion

In summary, unnatural death by undetermined suicide and fatal heroin overdoses were more highly correlated to previous contact with the Addiction Centre than were natural death, suicide, or violent death. Furthermore, there was a strong correlation between unnatural death by undetermined suicide and heroin overdoses on the one hand and additional substance use on the other, while suicide was not related to additional abuse.

## Competing interests

The authors declare that they have no competing interests.

## Authors' contributions

MB initiated and designed the study and was helpful in the drafting of the manuscript. PL initiated and designed the study and was helpful in the drafting of the manuscript. LB drafted the manuscript and contributed to the design. AL designed the statistical analysis. AF performed the staff interviews and read the case records.

## Pre-publication history

The pre-publication history for this paper can be accessed here:


